# Time to recurrence and BCLC stage at recurrence as critical variables in guiding treatment decisions for early-recurrent hepatocellular carcinoma after liver resection

**DOI:** 10.3389/fonc.2025.1672696

**Published:** 2025-10-03

**Authors:** Jian-Xi Zhang, Luo-Bin Guo, Chong-Shi Zeng, Qi-Zhen Huang, Zi-Sen Lai, Meng-Meng Wu, Qing-Jing Chen, Yong-Ping Lai, Xin-Feng Qiu, Bing Zhang, Jia-Cheng Zhang, Jia-Hui Lv, Li-Ming Huang, Wu-Yi You, Bin Wang, Kong-Ying Lin, Alfred Wei Chieh Kow, Yong-Yi Zeng

**Affiliations:** ^1^ Department of Hepatopancreatobiliary Surgery, First Affiliated Hospital of Fujian Medical University, Fuzhou, China; ^2^ Department of Hepatopancreatobiliary Surgery, Mengchao Hepatobiliary Hospital of Fujian Medical University, Fuzhou, China; ^3^ College of Science and Technology of China Three Gorges University, Yichang, China; ^4^ Department of Radiation Oncology, Mengchao Hepatobiliary Hospital of Fujian Medical University, Fuzhou, China; ^5^ Department of Hepatopancreatobiliary and Spleen Comprehensive Treatment, Mengchao Hepatobiliary Hospital of Fujian Medical University, Fuzhou, China; ^6^ Department of Radiation, Mengchao Hepatobiliary Hospital of Fujian Medical University, Fuzhou, China; ^7^ Department of Pathology, Mengchao Hepatobiliary Hospital of Fujian Medical University, Fuzhou, China; ^8^ Fujian Provincial Liver Disease Research Center, Fuzhou, China; ^9^ Division of Hepatobiliary and Pancreatic Surgery, Department of Surgery, National University Hospital, Singapore, Singapore

**Keywords:** hepatocellular carcinoma, liver resection, early recurrence, survival after recurrence, time to recurrence

## Abstract

**Background:**

Patients with Barcelona Clinic Liver Cancer (BCLC) stage 0/A hepatocellular carcinoma (HCC) represent the guideline-recommended population for liver resection; however, treatment strategies for early recurrence after resection remain debated. This study aimed to analyze prognostic factors for survival after recurrence (SAR) in patients with early recurrence following R0 resection of BCLC stage 0/A HCC and to develop evidence-based treatment recommendations integrating time to recurrence (TTR) and BCLC stage at recurrence.

**Methods:**

We conducted a retrospective review of 544 patients with early recurrence after R0 resection of BCLC stage 0/A HCC at a tertiary hepatopancreatobiliary academic hospital. Curative treatments included repeat liver resection and ablation, while non-curative treatments comprised transarterial chemoembolization and systemic therapy. Kaplan–Meier methods were applied to estimate SAR, and independent prognostic factors were identified with multivariable Cox regression analysis.

**Results:**

The median SAR was 39.4 months, with 1-year, 3-year, and 5-year SAR rates of 81.8%, 52.6%, and 39.0%, respectively. Patients receiving curative treatments demonstrated significantly improved SAR compared with those undergoing non-curative therapies (*P* < 0.001). Multivariable analysis identified TTR, alpha-fetoprotein level, albumin level, BCLC stage at recurrence, treatment modality, and microvascular invasion in initial tumors as independent prognostic factors for SAR. Subgroup analysis showed that integrating TTR and BCLC stage effectively guided treatment allocation: for BCLC stage A or C disease, treatment should follow current BCLC guidelines, whereas for stage B disease, curative therapy conferred survival benefit when TTR was >6 months but offered no benefit when TTR was ≤6 months.

**Conclusions:**

Curative treatments remain an effective option for selected patients with early-recurrent HCC. Treatment allocation based on TTR and BCLC stage at recurrence may optimize outcomes for this population.

## Graphical Abstract

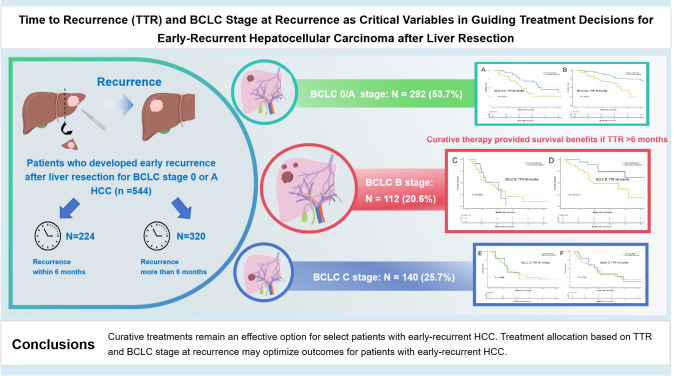

## Introduction

Hepatocellular carcinoma (HCC) is the sixth most prevalent malignancy worldwide and the third leading cause of cancer-related mortality (1). Liver resection remains the primary treatment recommended for patients with very early or early-stage HCC, specifically those classified as Barcelona Clinic Liver Cancer (BCLC) stage 0 or A (2). However, recurrence rates of 60% to 80% within five years after liver resection present significant challenges in clinical practice, substantially compromising long-term survival outcomes (3–5).

While staging and treatment guidelines—such as BCLC staging systems—are well-established for HCC, optimal management strategies for recurrent HCC remain highly debated (6–11). Recurrent HCC is typically classified by timing after resection: early recurrence (≤24 months) often reflects the aggressive nature of the initial tumor—characterized by high tumor burden, poor differentiation, microvascular invasion, and satellite nodules—whereas late recurrence (>24 months) generally represents *de novo* lesions with distinct biological features (12–14). Compared with late recurrence, early recurrence is not only more common—accounting for 56%–62% of cases—but is also associated with worse outcomes, highlighting the urgent need to refine treatment strategies for patients with early-recurrent HCC (14, 15).

Although previous studies have explored treatment options for recurrent HCC—including repeat resection, ablation, and salvage liver transplantation—these investigations have notable limitations. First, many studies aggregate early and late recurrence cases, overlooking important biological and prognostic distinctions between them, which may introduce bias and reduce the accuracy of treatment guidance (7, 10, 16). Furthermore, numerous studies include patients beyond BCLC stage 0/A, extending to stages B or C, where achieving R0 resection is less feasible and recurrence patterns and treatment responses differ markedly (3, 15, 17). This heterogeneity limits the applicability of current evidence to BCLC 0/A patients, who are the primary candidates for resection under existing treatment guidelines.

Therefore, we conducted a retrospective study to analyze recurrence patterns, survival after recurrence, and prognostic factors in patients with HCC initially staged as BCLC 0/A who experienced early recurrence following R0 resection. Additionally, we systematically evaluated the outcomes of various treatment modalities. Our aim was to provide clinicians with individualized therapeutic strategies to improve the long-term survival of patients with early-recurrent HCC.

## Methods

### Patients

This retrospective analysis reviewed data from 2,372 consecutive patients who underwent liver resection with curative intent for HCC at the Mengchao Hepatobiliary Hospital of Fujian Medical University between July 2014 and February 2022. Among these patients, 1,054 experienced early recurrence, defined as recurrence within 24 months postoperatively. Recurrent HCC was diagnosed based on pathology or clinical criteria, according to the American Association for the Study of Liver Diseases (18). Patients were included if they met the following criteria: (1) age at recurrence between 18 and 75 years; (2) initial tumor classified as BCLC stage 0 or A; (3) initial tumor had undergone R0 resection, confirmed by tumor-free surgical margins on postoperative pathology. Exclusion criteria were as follows: (1) patients who underwent liver transplantation for recurrent HCC, given the small sample size of this subgroup (n = 11); (2) severely impaired liver function or baseline condition at recurrence that restricted treatment options to best supportive care; (3) death within 30 days of recurrence diagnosis, to minimize bias in survival analysis due to treatment-related complications; and (4) incomplete clinical or follow-up data. This study was conducted in accordance with the Declaration of Helsinki and approved by the Ethics Committee of the Mengchao Hepatobiliary Hospital of Fujian Medical University. The requirement for written informed consent was waived because of the retrospective nature of the study.

### Clinical variables and definition

Data on demographics, early-recurrent tumor characteristics, post-recurrence treatments, and initial tumor features were systematically collected at the time of recurrence. Demographic variables included age, sex, history of liver disease, Child–Pugh grade, presence of cirrhosis, portal hypertension, serum albumin, total bilirubin, and alpha-fetoprotein (AFP) levels. Portal hypertension was defined as splenomegaly with thrombocytopenia (platelet count ≤100 × 10^9^/L) and/or the presence of esophageal varices. Tumor characteristics at recurrence included time to recurrence (TTR), imaging-based tumor size, tumor number, presence of macrovascular invasion, and extrahepatic metastasis. Post-recurrence treatments included ablation (radiofrequency or microwave), repeat resection, transarterial chemoembolization (TACE), radiotherapy, and systemic therapies. Initial tumor features were determined from pathology after the initial liver resection, including tumor size, tumor number, tumor encapsulation, microvascular invasion, satellite nodules, and tumor differentiation. Satellite nodules were defined as tumors <1 cm in diameter and located <1 cm from the main tumor. Tumor differentiation was graded using the Edmondson–Steiner system. Microvascular invasion was defined as clusters of tumor cells within endothelial-lined vascular spaces on microscopic examination. For patients with multiple tumors, tumor size was defined as the maximum diameter of the largest lesion.

### Treatment for early-recurrent HCC

Since 2014, our center has adopted a multidisciplinary team approach to guide the management of recurrent HCC, maintaining consistent treatment principles throughout the study period. Repeat liver resection is the preferred treatment for patients with preserved liver function, adequate residual liver volume, and recurrences not involving major vessels, with the goal of achieving curative outcomes. In cases of macrovascular invasion, repeat liver resection may be considered if en-bloc resection is feasible. Repeat liver resection is also considered for patients with isolated, resectable extrahepatic metastases confirmed by multimodal imaging. Palliative liver resection is not routinely performed at our center, except in emergencies requiring urgent surgical intervention. Ablation is preferred for patients with a single intrahepatic lesion ≤3 cm, or up to three lesions each ≤3 cm, who are unsuitable for or unwilling to undergo repeat hepatectomy. In selected patients, repeat liver resection combined with ablation is performed. TACE is employed when resection is not feasible and there is no vascular or extrahepatic involvement. Systemic therapies are administered to patients with vascular invasion or extrahepatic metastases, often in combination with TACE or radiotherapy to enhance intrahepatic tumor control, provided liver function is adequate. Supportive care is offered to patients with extensive metastases or severely impaired liver function.

### Follow-up

The follow-up schedule for early-recurrent HCC was designed according to the therapeutic approach. Patients who received curative treatments (repeat hepatectomy or ablation) underwent follow-up every two months during the first two years and every 3–6 months thereafter if no recurrence was detected. For those who received TACE or systemic therapy, follow-up was adjusted according to tumor control, typically beginning with an assessment one month after treatment and continuing every two months. Standard follow-up assessments included physical examination, liver function tests, AFP monitoring, abdominal ultrasound, and contrast-enhanced computed tomography or magnetic resonance imaging. If tumor recurrence or progression was detected, treatment options such as repeat liver resection, ablation, TACE, systemic therapy, or radiotherapy were selected based on tumor characteristics, performance status, liver function reserve, and patient preference.

The primary endpoint of this study was survival after recurrence (SAR), defined as the time from the diagnosis of early-recurrent HCC to death or last follow-up.

### Statistics

Continuous variables are reported as mean ± standard deviation or median (interquartile range) and compared using Student’s t-test or the Mann-Whitney U test. Categorical variables are presented as frequencies (percentages) and analyzed with chi-square or Fisher’s exact tests. Kaplan-Meier methods were used to plot SAR curves, with comparisons between groups tested using the log-rank test. To enhance clinical applicability, an optimal prognostic cutoff for TTR was further identified using survival decision trees. Cox proportional hazards regression analysis was performed to identify independent factors associated with SAR, with variables with *P* < 0.05 in univariable analysis included in the multivariable Cox model. All statistical analyses were conducted using SPSS version 20 (SPSS, Inc., Chicago, IL, USA) and R version 4.1.1 (R Project, Vienna, Austria).

## Results

A total of 544 patients who experienced early recurrence after liver resection for HCC initially staged as BCLC 0/A were included in this study (**Supplementary Figure 1**). As shown in **Table 1**, most patients had hepatitis B virus infection (95.4%) and cirrhosis (80.9%), while 95 patients (17.5%) presented with portal hypertension. The median diameter of the initial tumor was 4.9 cm (interquartile range [IQR], 2.8–9.0 cm), with multiple tumors observed in 5.5% of cases (n = 30). Microvascular invasion was identified in 78.5% of cases (n = 427), satellite nodules in 24.8% (n = 135), incomplete tumor capsule in 69.7% (n = 379), and poor differentiation in 68.8% (n = 374).

**Table 1:** Baseline characteristics of patients.

Variables	All
	(N=544)
Age, years, mean (SD)	55.8 ± 11.3
Gender, male	474 (87.1%)
HBV infection	519 (95.4%)
Cirrhosis	440 (80.9%)
Portal hypertension	95 (17.5%)
Child-Pugh grade A	489 (89.9%)
Albumin, g/L, mean (SD)	36.4 ± 5.4
Total bilirubin, umol/L, median (IQR)	15.0 (11.8, 20.0)
AFP> 400 ng/mL	123 (22.6%)
Time to recurrence, month, median (IQR)	7.4 (4.2, 13.0)
Multiple tumors	306 (56.3%)
Tumor size, cm, median (IQR)	1.6 (1.1, 3.0)
Macrovascular invasion	37 (6.8%)
Extrahepatic recurrence	111 (20.4%)
Lung	58 (10.7%)
Abdomen	34 (6.3%)
Bone	5 (0.9%)
Brain	3 (0.6%)
Multiple organ	11 (2.0%)
BCLC staging system	
0/A	292 (53.7%)
B	112 (20.6%)
C	140 (25.7%)
Curative treatment for recurrence	238 (43.8%)
Initial tumor characteristics	
Tumor size, cm, median (IQR)	4.9 (2.8, 9.0)
Multiple tumors	30 (5.5%)
Incomplete Tumor capsule	379 (69.7%)
Poor differentiation	374 (68.8%)
Satellite nodules	135 (24.8%)
Microvascular invasion	427 (78.5%)

Values are n (%), mean ± standard deviation, or median (interquartile range).

*AFP*, α-fetoprotein; *BCLC*, Barcelona Clinic Liver Cancer; *HBV*, hepatitis B virus; *IQR*, interquartile range; *SD*, standard deviation.

### Recurrence patterns of early-recurrent HCC

**Figure 1:**
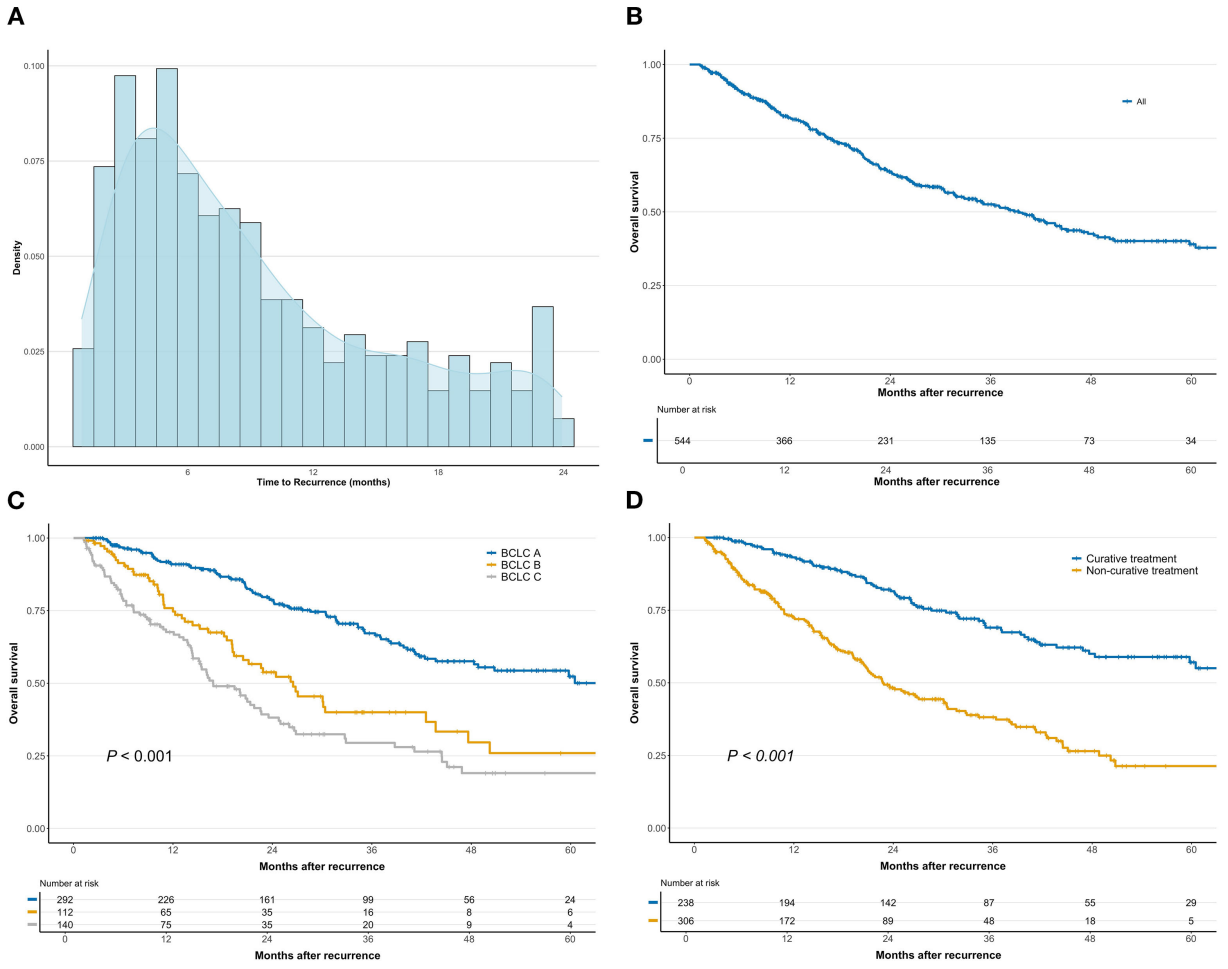
Distribution of time to recurrence **(A)**, survival after recurrence **(B)**, survival after recurrence by Barcelona Clinic Liver Cancer (BCLC) staging system **(C)**, and treatment modalities for recurrence **(D)** in patients with early recurrence following liver resection for hepatocellular carcinoma.

The median TTR was 7.4 months (IQR, 4.2–13.0) (**Figure 1A**). At recurrence, the median tumor diameter was 1.6 cm (IQR, 1.1–3.0 cm), with 306 patients (56.3%) having multiple lesions (**Table 1**). Macrovascular invasion was identified in 37 patients (6.8%), and 111 patients (20.4%) had extrahepatic metastases, with metastatic sites including the lung (n = 58, 10.7%), abdominal cavity (n = 34, 6.3%), bone (n = 5, 0.9%), brain (n = 3, 0.6%), and multiple organs (n = 11, 2.0%). By BCLC stage at recurrence, 292 patients (53.7%) were stage 0/A, 112 (20.6%) were stage B, and 140 (25.7%) were stage C. Regarding treatment for recurrence, 238 patients (43.8%) received curative therapy, including repeat hepatectomy (n = 121, 22.2%) and ablation (n = 117, 21.5%). Meanwhile, 187 patients (34.3%) underwent TACE, and 119 (21.9%) received systemic therapy or a combination of systemic and local therapies (**Table 1**).

### Survival after recurrence

The median follow-up time after recurrence was 20.1 months (range, 1.2–98.2 months). The median SAR was 39.4 months (95% confidence interval [CI], 32.9–45.1), with 1-, 3-, and 5-year SAR rates of 81.8%, 52.6%, and 39.0%, respectively (**Figure 1B**). SAR was significantly associated with BCLC stage at recurrence. The median SAR and 5-year SAR rates were 83.3 months and 52.3% for stage 0/A, 26.5 months and 25.9% for stage B, and 16.8 months and 19.0% for stage C (*P* < 0.001, **Figure 1C**). Patients who underwent curative treatment had significantly longer SAR than those who received non-curative treatment, with a median SAR of 75.0 months and a 5-year SAR rate of 57.1% versus 22.7 months and 21.3%, respectively (*P* < 0.001, **Figure 1D**).

### Independent factors for SAR

Multivariable Cox regression analysis identified longer TTR (hazard ratio [HR], 0.972; 95% confidence interval [CI], 0.948–0.997; *P* = 0.030), higher AFP level at recurrence (HR, 1.496; 95% CI, 1.098–2.038; *P* = 0.011), lower serum albumin level at recurrence (HR, 1.452; 95% CI, 1.083–1.946; *P* = 0.013), more advanced BCLC stage at recurrence (HRs, 1.638 and 2.042; 95% CIs, 1.130–2.373 and 1.432–2.912; *P* = 0.009 and *P* < 0.001, respectively), receipt of non-curative treatment for recurrence (HR, 1.987; 95% CI, 1.433–2.756; *P* < 0.001), and microvascular invasion in the initial tumor (HR, 1.465; 95% CI, 1.039–2.064; *P* = 0.029) as independent prognostic factors for SAR (**Table 2**).

**Table 2:** Univariable and multivariable analysis of risk factors associated with SAR.

Characteristics	HR comparison	UV HR (95% CI)	UV *P*	MV HR (95% CI)	MV *P*
Age	> 55 *vs*. ≤ 55 years	0.897 (0.691-1.164)	0.413		
Gender	Male *vs.* female	1.489 (0.950-2.333)	0.082		
HBV infection	Yes *vs.* no	1.165 (0.635-2.136)	0.622		
Cirrhosis	Yes *vs*. no	0.985 (0.692-1.401)	0.931		
Child-Pugh grade	B *vs*. A	1.605 (1.104-2.334)	0.013	*NS*	0.634
Portal hypertension	Yes *vs.* no	0.795 (0.554-1.140)	0.212		
Albumin	< 35 *vs.* ≥ 35 g/L	1.342 (1.031-1.747)	0.029	1.452 (1.083-1.946)	0.013
Total bilirubin	> 17.1 *vs.* ≤ 17.1 umol/L	1.187 (0.913-1.543)	0.201		
AFP	> 400 *vs.* ≤ 400 ng/ml	2.333 (1.762-3.088)	< 0.001	1.496 (1.098-2.038)	0.011
Time to recurrence	Per months	0.938 (0.915-0.961)	< 0.001	0.972 (0.948-0.997)	0.030
BCLC	B *vs*. A	2.303 (1.637-3.240)	< 0.001	1.638 (1.130-2.373)	0.009
BCLC	C *vs*. A	3.358 (2.490-4.529)	< 0.001	2.042 (1.432-2.912)	< 0.001
Treatment for recurrence	Non-curative *vs*. curative	2.971 (2.231-3.956)	< 0.001	1.987 (1.433-2.756)	< 0.001
Tumor size (initial tumor)	> 5 *vs*. ≤ 5 cm	1.383 (1.064-1.797)	0.015	*NS*	0.338
Tumor number (initial tumor)	Multiple *vs*. single	1.491 (0.932-2.386)	0.096		
Tumor encapsulation (initial tumor)	Incomplete *vs*. complete	1.353 (0.981-1.866)	0.065		
Tumor differentiation (initial tumor)	Poor *vs*. well	1.167 (0.885-1.541)	0.274		
Satellite nodules (initial tumor)	Yes *vs*. no	1.433 (1.084-1.893)	0.011	*NS*	0.183
Microvascular invasion (initial tumor)	Yes *vs*. no	1.578 (1.132-2.200)	0.007	1.465 (1.039-2.064)	0.029

*AFP*, α-fetoprotein; *BCLC*, Barcelona Clinic Liver Cancer; *CI*, confidence interval; *HBV*, hepatitis B virus; *HR*, Hazard ratio; *MV*, multivariable; *NS*, not significant; *SAR*, survival after recurrence; *UV*, univariable.

NS, not significant.

### Association of TTR with recurrence patterns of early-recurrent HCC and SAR

**Figure 2:**
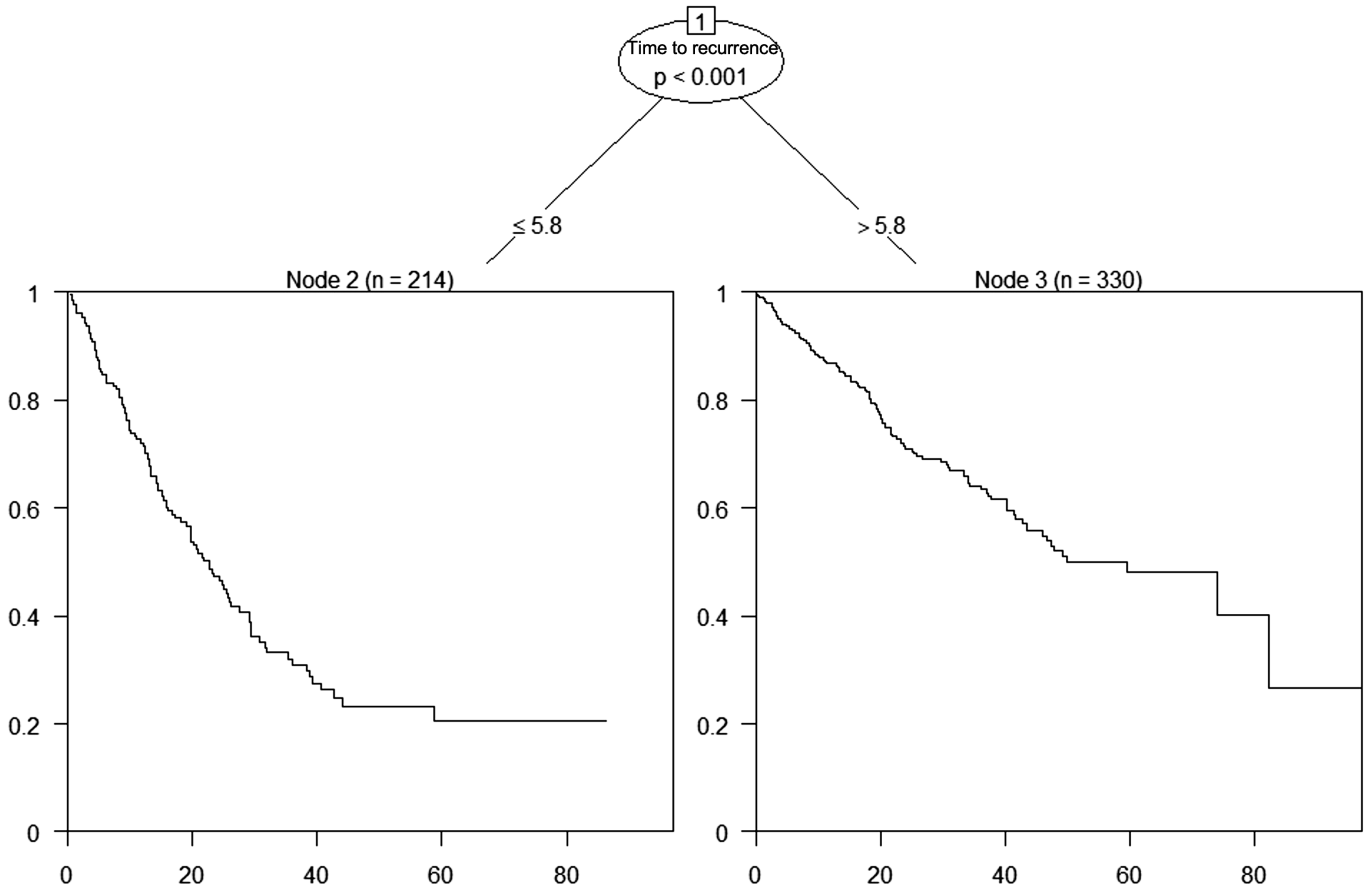
Decision tree analysis to determine the optimal cutoff value of time to recurrence for survival after recurrence.

**Figure 3:**
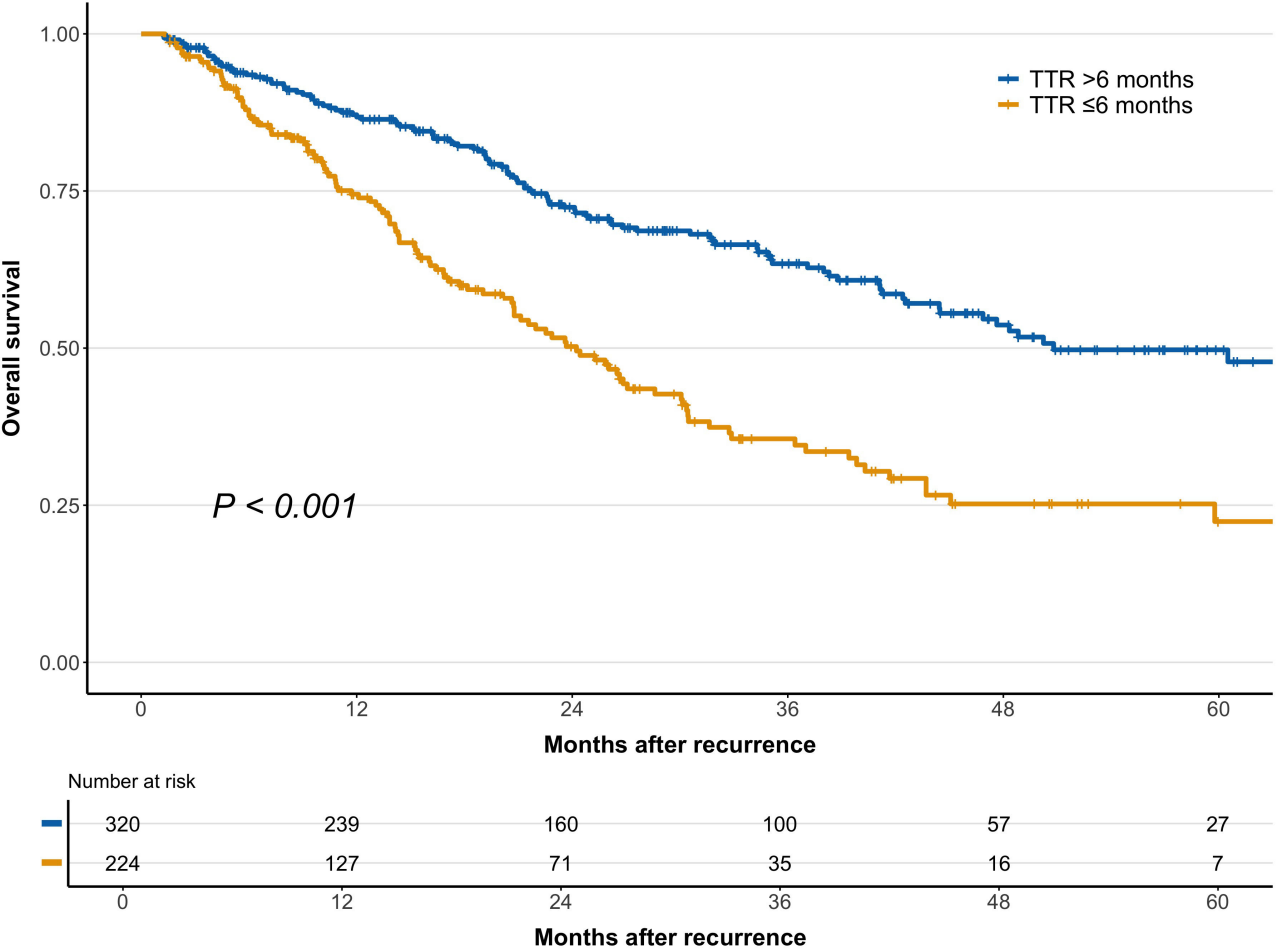
Comparison of survival after recurrence between patients with time to recurrence (TTR) ≥6 months and TTR <6 months.

Decision tree analysis identified an optimal TTR cutoff of 5.8 months for SAR, which was rounded to 6 months for clinical applicability (**Figure 2**). As shown in **Table 3**, patients with TTR ≤6 months had significantly higher rates of multiple tumors, extrahepatic metastases, and advanced BCLC stage at recurrence (all *P* < 0.001) and were less likely to receive curative treatment (*P* < 0.001). Survival analysis demonstrated that patients with TTR ≤6 months had significantly worse SAR than those with TTR >6 months (**Figure 3**), a trend that remained consistent across BCLC stages and treatment modalities (**Supplementary Figure 2**). Multivariable Cox regression further confirmed TTR ≤6 months as an independent risk factor for SAR (**Supplementary Table 1**).

**Table 3:** Comparison of baseline characteristics between patients with TTR of ≥6 months and <6 months.

Variables	TTR ≤ 6 months	TTR > 6 months	P-value
	(N=224)	(N=320)	
Age, years, mean (SD)	55.0 ± 12.3	56.5 ± 10.6	0.143
Gender, male	192 (85.7%)	282 (88.1%)	0.409
HBV infection	215 (96.0%)	304 (95.0%)	0.590
Cirrhosis	176 (78.6%)	264 (82.5%)	0.251
Portal hypertension	31 (13.8%)	64 (20.0%)	0.063
Child-Pugh grade A	200 (89.3%)	289 (90.3%)	0.696
Albumin, g/L, mean (SD)	36.3 ± 5.2	36.5 ± 5.5	0.601
Total bilirubin, umol/L, median (IQR)	14.4 (11.3, 19.3)	15.2 (12.4, 20.2)	0.443
AFP> 400 ng/mL	70 (31.3%)	53 (16.6%)	< 0.001
Multiple tumors	145 (64.7%)	161 (50.3%)	< 0.001
Tumor size, cm, median (IQR)	1.7 (1.0, 3.2)	1.6 (1.1, 2.9)	0.823
Macrovascular invasion	15 (6.7%)	22 (6.9%)	0.935
Extrahepatic recurrence	68 (30.4%)	43 (13.4%)	< 0.001
Lung	35 (15.6%)	23 (7.2%)	< 0.001
Abdomen	21 (9.4%)	13 (4.1%)	
Bone	4 (1.8%)	1 (0.3%)	
Brain	2 (0.9%)	1 (0.3%)	
Multiple organ	6 (2.7%)	5 (1.6%)	
BCLC staging system			
0/A	91 (40.6%)	201 (62.8%)	< 0.001
B	54 (24.1%)	58 (18.1%)	
C	79 (35.3%)	61 (19.1%)	
Curative treatment for recurrence	63 (28.1%)	175 (54.7%)	< 0.001
Initial tumor characteristics			
Tumor size, cm, median (IQR)	6.5 (3.2, 10.0)	4.0 (2.5, 7.0)	< 0.001
Multiple tumors	13 (5.8%)	17 (5.3%)	0.805
Incomplete Tumor capsule	154 (68.8%)	225 (70.3%)	0.696
Poor differentiation	152 (67.9%)	222 (69.4%)	0.707
Satellite nodules	76 (33.9%)	59 (18.4%)	< 0.001
Microvascular invasion	191 (85.3%)	236 (73.8%)	0.001

Values are n (%), mean ± standard deviation, or median (interquartile range).

*AFP*, α-fetoprotein; *BCLC*, Barcelona Clinic Liver Cancer; *HBV*, hepatitis B virus; *IQR*, interquartile range; *SD*, standard deviation; *TTR*, time to recurrence.

### Role of TTR and BCLC stage at recurrence in treatment decision-making

**Figure 4:**
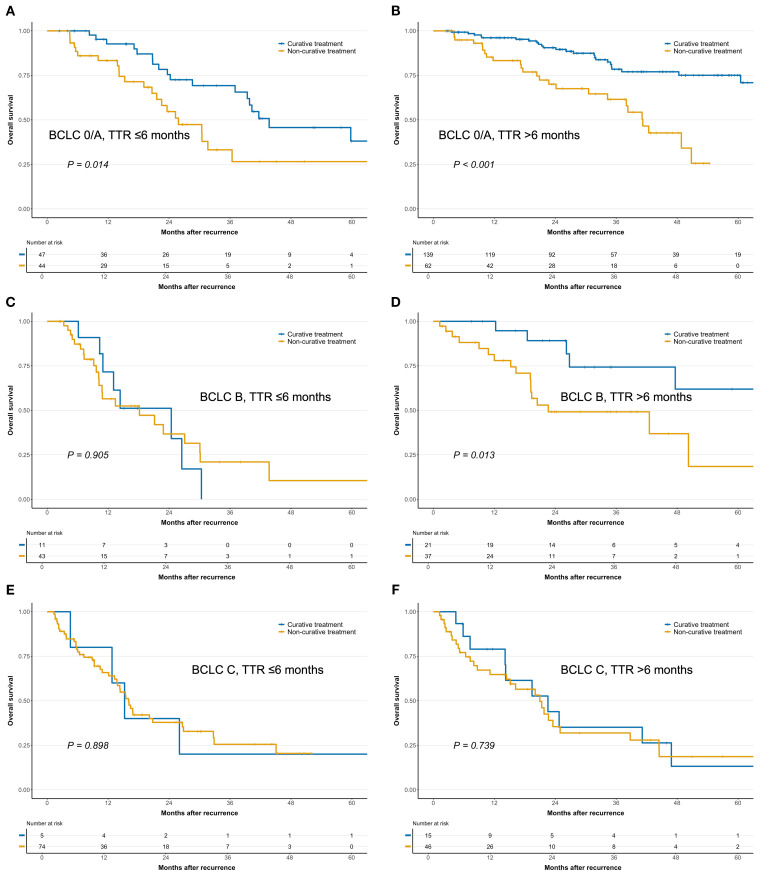
Comparison of survival after recurrence between curative and non-curative treatments in patients with early-recurrent hepatocellular carcinoma (HCC), stratified by Barcelona Clinic Liver Cancer (BCLC) stage at recurrence and time to recurrence (TTR). **(A)** BCLC stage A and TTR ≤6 months; **(B)** BCLC stage A and TTR >6 months; **(C)** BCLC stage B and TTR ≤6 months; **(D)** BCLC stage B and TTR >6 months; **(E)** BCLC stage C and TTR ≤6 months; **(F)** BCLC stage C and TTR >6 months.

Further analysis of SAR across treatment approaches, stratified by TTR and BCLC stage at recurrence, indicated that these factors can effectively guide treatment allocation (**Figure 4**). Among patients with BCLC stage 0/A at recurrence, both those with TTR >6 months and those with TTR ≤6 months achieved significantly better SAR with curative treatments than with non-curative options (*P* < 0.05; **Figures 4A, B**). In contrast, curative treatment conferred no survival benefit in patients with BCLC stage B and TTR ≤6 months, or in those with BCLC stage C at recurrence (**Figures 4C–F**).

## Discussion

Treatment of recurrent HCC remains challenging, particularly for early-recurrent disease, which is frequently associated with shorter SAR. In this study, we analyzed a large cohort of patients with BCLC stage 0/A HCC who experienced early recurrence after curative resection. Our results demonstrated that SAR in early-recurrent HCC was associated with TTR, serum albumin and AFP levels at recurrence, BCLC stage at recurrence, treatment modality, and microvascular invasion in the initial tumor. Furthermore, we propose treatment recommendations for early-recurrent HCC stratified by TTR and BCLC stage at recurrence. These findings may provide valuable clinical insights for managing early recurrence in patients with BCLC stage 0/A HCC, who represent the optimal candidates for liver resection under current guidelines.

Curative interventions, including ablation, repeat liver resection, and salvage liver transplantation, can substantially improve outcomes in selected patients with recurrent HCC (7, 19–22). In this study of patients with BCLC stage 0/A HCC who experienced early recurrence after hepatectomy, the 1-, 3-, and 5-year SAR rates were 81.8%, 52.6%, and 39.0%, respectively. Patients who received curative treatments had significantly higher SAR than those who underwent non-curative therapies, underscoring the central role of aggressive curative approaches in improving prognosis. These findings align with previous studies demonstrating the safety and feasibility of curative therapies for recurrent HCC, particularly in the context of advances in surgical techniques and perioperative care (6, 7, 19, 21, 23). These findings further emphasize the importance of routine postoperative follow-up to enable early detection and timely curative interventions (24, 25). In this study, 43.8% of patients underwent curative therapy, reflecting our center’s emphasis on comprehensive, long-term patient care. As a specialized hepatopancreatobiliary academic hospital, we implemented a cloud-based follow-up system and established a dedicated health management team. These initiatives play a vital role in out-of-hospital patient management, ensuring timely follow-up and improving the detection and management of early recurrence, thereby maximizing opportunities for curative intervention.

Management of HCC remains challenging because of its heterogeneity and the complex interplay of numerous factors influencing prognosis and treatment. This study highlights the prognostic significance of BCLC stage at recurrence and TTR in patients with early-recurrent HCC. The BCLC staging system, a cornerstone of prognostic stratification and treatment guidance in HCC, was applied to early-recurrent disease in this study, underscoring its value in guiding both prognosis and treatment. TTR, a critical factor distinguishing early from late recurrence, is also a well-established prognostic indicator in recurrent HCC (24, 26). Our findings confirm its prognostic relevance in early-recurrent HCC. In this study, patients with TTR ≤6 months had a higher prevalence of multiple tumors, extrahepatic metastases, and more advanced BCLC stage at recurrence. Multivariable analysis further demonstrated that patients with TTR ≤6 months had significantly shorter SAR than those with TTR >6 months. Early recurrence likely reflects micrometastatic dissemination from the initial tumor. We suggest that a shorter TTR may indicate more aggressive biological behavior of micrometastases, whereas a longer TTR may reflect relatively indolent biological characteristics. However, further mechanistic studies are needed to validate this hypothesis.

**Figure 5:**
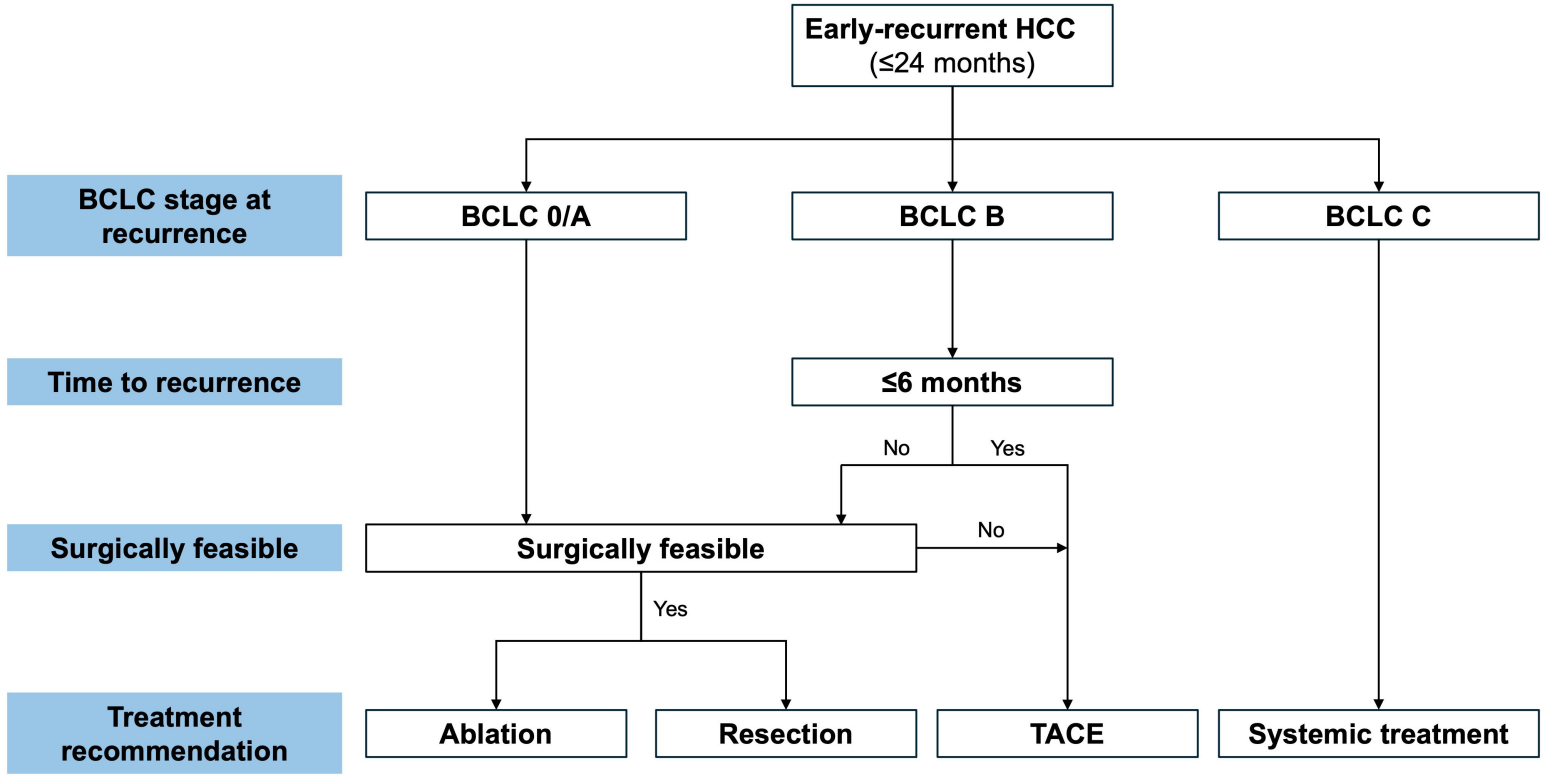
Recommendations for treatment decision making for early-recurrent hepatocellular carcinoma (HCC) based on Barcelona Clinic Liver Cancer (BCLC) stage at recurrence and time to recurrence. TACE, transarterial chemoembolization.

Importantly, this study is, to our knowledge, the first to demonstrate that combining BCLC stage with TTR can guide prognosis-based treatment strategies for early-recurrent HCC (**Figure 5**). Our results suggest that treatment decisions for patients with early-recurrent HCC at BCLC stage A or C should adhere to current BCLC guidelines. However, among patients with early recurrence at BCLC stage B, TTR emerged as a decisive factor: curative treatment conferred survival benefit when TTR was >6 months but provided no benefit when TTR was ≤6 months (**Figure 5**). These findings warrant further investigation to validate their broader applicability.

Additionally, our study indicates that AFP levels at recurrence and MVI in the initial tumor significantly influence SAR. Elevated AFP levels and MVI are indicative of aggressive tumor biology (27). Considering these variables during treatment planning may enhance prognostic accuracy and support individualized therapeutic approaches. High-risk patients may benefit from comprehensive perioperative management strategies (4). Emerging evidence suggests that neoadjuvant systemic therapies, including immune checkpoint inhibitors and targeted agents, may reduce tumor burden and eradicate micrometastatic disease, potentially decreasing recurrence rates and enhancing survival outcomes (28–30). Although the benefit of adjuvant systemic therapy remains uncertain, its potential role in recurrence prevention—particularly in patients with aggressive tumor biology, such as those with MVI—warrants further investigation (31). Prospective trials are necessary to determine the clinical utility of these strategies in improving survival outcomes in early-recurrent HCC.

This study has several limitations. First, as a single-center retrospective study, the generalizability of our findings requires validation by multicenter studies. However, as a tertiary academic hospital specializing in hepatopancreatobiliary surgery, our single-center cohort ensured consistency in treatment protocols, which adhered to current HCC guideline recommendations, enhancing the reliability of our results. Second, the cohort is derived from a region with a high prevalence of hepatitis B, and the applicability of these findings should be confirmed in populations where hepatitis C and alcohol-related liver disease are more prevalent, such as in Western countries. Third, although our study described key recurrence-related characteristics—including intrahepatic versus extrahepatic recurrence and vascular invasion—we were unable to differentiate between surgical marginal recurrences and distal intrahepatic recurrences due to lack of detailed spatial data in the current database. We acknowledge that this distinction may reflect different biological behaviors and influence treatment strategies, and future prospective studies with more granular anatomical recurrence data are warranted. Finally, we excluded patients with early-recurrent HCC who underwent salvage liver transplantation due to small sample sizes. Our center only gained transplant qualifications at the end of 2023, and patients who received salvage transplants at external institutions were excluded due to small sample size or incomplete clinical and follow-up data. Given that liver transplantation remains the most effective curative treatment for HCC, further research is warranted to evaluate its role and prognosis in early-recurrent HCC.

## Conclusion

In conclusion, curative treatment remains an effective option for select patients with early-recurrent HCC. A combined evaluation of TTR and BCLC staging at recurrence may inform treatment decisions, providing a novel approach to enhance long-term outcomes. Further studies are needed to validate these findings and support more individualized, evidence-based treatment strategies for patients with early-recurrent HCC.

## Data Availability

The original contributions presented in the study are included in the article/**Supplementary Material**. Further inquiries can be directed to the corresponding authors.

## References

[1] Sung H Ferlay J Siegel RL Laversanne M Soerjomataram I Jemal A. Global cancer statistics 2020: GLOBOCAN estimates of incidence and mortality worldwide for 36 cancers in 185 countries. CA Cancer J Clin. (2021) 71:209–49. doi: 10.3322/caac.21660, PMID: 33538338

[2] Reig M Forner A Rimola J Ferrer-Fàbrega J Burrel M Garcia-Criado Á. BCLC strategy for prognosis prediction and treatment recommendation: the 2022 update. J Hepatol. (2022) 76:681–93. doi: 10.1016/j.jhep.2021.11.018, PMID: 34801630 PMC8866082

[3] Tabrizian P Jibara G Shrager B Schwartz M Roayaie S. Recurrence of hepatocellular cancer after resection: patterns, treatments, and prognosis. Ann Surg. (2015) 261:947–55. doi: 10.1097/SLA.0000000000000710, PMID: 25010665

[4] Llovet JM Pinyol R Yarchoan M Singal AG Marron TU Schwartz M. Adjuvant and neoadjuvant immunotherapies in hepatocellular carcinoma. Nat Rev Clin Oncol. (2024) 21:294–311. doi: 10.1038/s41571-024-00868-0, PMID: 38424197 PMC11984461

[5] Bodzin AS Lunsford KE Markovic D Harlander-Locke MP Busuttil RW Agopian VG. Predicting mortality in patients developing recurrent hepatocellular carcinoma after liver transplantation: impact of treatment modality and recurrence characteristics. Ann Surg. (2017) 266:118–25. doi: 10.1097/SLA.0000000000001894, PMID: 27433914

[6] He W Peng B Tang Y Yang J Zheng Y Qiu J. Nomogram to predict survival of patients with recurrence of hepatocellular carcinoma after surgery. Clin Gastroenterol Hepatol Off Clin Pract J Am Gastroenterol Assoc. (2018) 16:756–764.e10. doi: 10.1016/j.cgh.2017.12.002, PMID: 29246702

[7] Yoh T Seo S Taura K Iguchi K Ogiso S Fukumitsu K. Surgery for recurrent hepatocellular carcinoma: achieving long-term survival. Ann Surg. (2021) 273:792–9. doi: 10.1097/SLA.0000000000003358, PMID: 31058698

[8] Chua DW Koh YX Syn NL Chuan TY Yao TJ Lee SY. Repeat hepatectomy versus radiofrequency ablation in management of recurrent hepatocellular carcinoma: an average treatment effect analysis. Ann Surg Oncol. (2021) 28:7731–40. doi: 10.1245/s10434-021-09948-2, PMID: 33969464

[9] Guo Y Ren Y Chen L Sun T Zhang W Sun B. Transarterial chemoembolization combined with camrelizumab for recurrent hepatocellular carcinoma. BMC Cancer. (2022) 22:270. doi: 10.1186/s12885-022-09325-6, PMID: 35287627 PMC8922827

[10] Yoon YI Song GW Lee S Moon D Hwang S Kang WH. Salvage living donor liver transplantation versus repeat liver resection for patients with recurrent hepatocellular carcinoma and Child-Pugh class A liver cirrhosis: A propensity score-matched comparison. Am J Transplant. (2022) 22:165–76. doi: 10.1111/ajt.16790, PMID: 34383368

[11] Zhang X Li C Wen T Peng W Yan L Yang J. Treatment for intrahepatic recurrence after curative resection of hepatocellular carcinoma: Salvage liver transplantation or re-resection/radiofrequency ablation? A Retrospective Cohort Study. Int J Surg. (2017) 46:178–85. doi: 10.1016/j.ijsu.2017.09.001, PMID: 28890407

[12] Xu XF Xing H Han J Li ZL Lau WY Zhou YH. Risk factors, patterns, and outcomes of late recurrence after liver resection for hepatocellular carcinoma: a multicenter study from China. JAMA Surg. (2019) 154:209–17. doi: 10.1001/jamasurg.2018.4334, PMID: 30422241 PMC6439634

[13] Imamura H Matsuyama Y Tanaka E Ohkubo T Hasegawa K Miyagawa S. Risk factors contributing to early and late phase intrahepatic recurrence of hepatocellular carcinoma after hepatectomy. J Hepatol. (2003) 38:200–7. doi: 10.1016/s0168-8278(02)00360-4, PMID: 12547409

[14] Wang MD Li C Liang L Xing H Sun LY Quan B. Early and late recurrence of hepatitis B virus-associated hepatocellular carcinoma. Oncologist. (2020) 25:e1541–51. doi: 10.1634/theoncologist.2019-0944, PMID: 32472951 PMC7543359

[15] Yagi R Midorikawa Y Moriguchi M Nakayama H Aramaki O Yamazaki S. Liver resection for recurrent hepatocellular carcinoma to improve survivability: a proposal of indication criteria. Surgery. (2018) 163:1250–6. doi: 10.1016/j.surg.2017.12.022, PMID: 29452700

[16] Xia Y Li J Liu G Wang K Qian G Lu Z. Long-term effects of repeat hepatectomy vs percutaneous radiofrequency ablation among patients with recurrent hepatocellular carcinoma: A randomized clinical trial. JAMA Oncol. (2020) 6:255. doi: 10.1001/jamaoncol.2019.4477, PMID: 31774468 PMC6902111

[17] Shindoh J Matsumura M Kobayashi M Akabane M Okubo S Hashimoto M. Disease-free interval and tumor stage complementarily predict the biological behavior of recurrent hepatocellular carcinoma. Ann Surg Oncol. (2023) 30:3402–10. doi: 10.1245/s10434-023-13228-6, PMID: 36808590

[18] Marrero JA Kulik LM Sirlin CB Zhu AX Finn RS Abecassis MM. Diagnosis, staging, and management of hepatocellular carcinoma: 2018 practice guidance by the american association for the study of liver diseases. Hepatol Baltim Md. (2018) 68:723–50. doi: 10.1002/hep.29913, PMID: 29624699

[19] Yamashita Y Shirabe K Tsuijita E Takeishi K Ikegami T Yoshizumi T. Third or more repeat hepatectomy for recurrent hepatocellular carcinoma. Surgery. (2013) 154:1038–45. doi: 10.1016/j.surg.2013.04.046, PMID: 23973109

[20] Yuan BH Zhu YK Zou XM Zhou HD Li RH Zhong JH. Repeat hepatic resection versus percutaneous ablation for the treatment of recurrent hepatocellular carcinoma: meta-analysis. BJS Open. (2022) 6:zrac036. doi: 10.1093/bjsopen/zrac036, PMID: 35482024 PMC9048940

[21] Mise Y Hasegawa K Shindoh J Ishizawa T Aoki T Sakamoto Y. The feasibility of third or more repeat hepatectomy for recurrent hepatocellular carcinoma. Ann Surg. (2015) 262:347–57. doi: 10.1097/SLA.0000000000000882, PMID: 25185473

[22] Liu J Zhang G Yang L Yan D Yu J Wei S. Salvage liver transplantation versus curative treatment for patients with recurrent hepatocellular carcinoma: A systematic review and meta-analysis. Eur J Surg Oncol J Eur Soc Surg Oncol Br Assoc Surg Oncol. (2024) 50:108427. doi: 10.1016/j.ejso.2024.108427, PMID: 38796968

[23] Erridge S Pucher PH Markar SR Malietzis G Athanasiou T Darzi A. Meta-analysis of determinants of survival following treatment of recurrent hepatocellular carcinoma. Br J Surg. (2017) 104:1433–42. doi: 10.1002/bjs.10597, PMID: 28628947

[24] Yao LQ Chen ZL Feng ZH Diao YK Li C Sun HY. Clinical features of recurrence after hepatic resection for early-stage hepatocellular carcinoma and long-term survival outcomes of patients with recurrence: a multi-institutional analysis. Ann Surg Oncol. (2022) . doi: 10.1245/s10434-022-11454-y, PMID: 35192156

[25] Yan WT Li C Yao LQ Qiu HB Wang MD Xu XF. Predictors and long-term prognosis of early and late recurrence for patients undergoing hepatic resection of hepatocellular carcinoma: a large-scale multicenter study. Hepatobiliary Surg Nutr. (2023) 12:155–68. doi: 10.21037/hbsn-21-288, PMID: 37124678 PMC10129892

[26] Moazzam Z Alaimo L Endo Y Lima HA Woldesenbet S Rueda BO. A prognostic model to predict survival after recurrence among patients with recurrent hepatocellular carcinoma. Ann Surg. (2024) 279:471–8. doi: 10.1097/SLA.0000000000006056, PMID: 37522251

[27] Meniconi RL Komatsu S Perdigao F Boëlle PY Soubrane O Scatton O. Recurrent hepatocellular carcinoma: a western strategy that emphasizes the impact of pathologic profile of the first resection. Surgery. (2015) 157:454–62. doi: 10.1016/j.surg.2014.10.011, PMID: 25633732

[28] Marron TU Fiel MI Hamon P Fiaschi N Kim E Ward SC Neoadjuvant cemiplimab for resectable hepatocellular carcinoma: a single-arm, open-label, phase 2 trial. Lancet Gastroenterol Hepatol. (2022) 7:219–29. doi: 10.1016/S2468-1253(21)00385-X, PMID: 35065058 PMC9901534

[29] Xia Y Tang W Qian X Li X Cheng F Wang K. Efficacy and safety of camrelizumab plus apatinib during the perioperative period in resectable hepatocellular carcinoma: a single-arm, open label, phase II clinical trial. J Immunother Cancer. (2022) 10:e004656. doi: 10.1136/jitc-2022-004656, PMID: 35379737 PMC8981365

[30] Cao Y Tang H Hu B Zhang W Wan T Han J. Comparison of survival benefit between salvage surgery after conversion therapy versus surgery alone for hepatocellular carcinoma with portal vein tumor thrombosis: a propensity score analysis. HPB. (2023) 25:775–87. doi: 10.1016/j.hpb.2023.03.004, PMID: 36973160

[31] Wang K Xiang YJ Yu HM Cheng YQ Liu ZH Qin YY. Adjuvant sintilimab in resected high-risk hepatocellular carcinoma: a randomized, controlled, phase 2 trial. Nat Med. (2024) 30:708–15. doi: 10.1038/s41591-023-02786-7, PMID: 38242982

