# Supplementary material for: Time to recurrence and BCLC stage at recurrence as critical variables in guiding treatment decisions for early-recurrent hepatocellular carcinoma after liver resection
Source: Front Oncol. 2025 Oct 3;15:1672696. doi: 10.3389/fonc.2025.1672696 (PMC12532004; doi:10.3389/fonc.2025.1672696)

**Time to Recurrence and BCLC Stage at Recurrence as Critical Variables in Guiding Treatment Decisions for Early-Recurrent Hepatocellular Carcinoma after Liver Resection**

Jian-Xi Zhang *MD*^1,2*^, Luo-Bin Guo *MD*^2*^, Chong-Shi Zeng *MD*^2,3*^, Qi-Zhen Huang *MD*^4*^, Zi-Sen Lai *MD*^2^, Meng-Meng Wu *MD*^2^, Qing-Jing Chen *MD*^2^, Yong-Ping Lai *MD*^2^, Xin-Feng Qiu *MD*^2^, Bing Zhang *MD*^5^, Jia-Cheng Zhang *MD*^2^, Jia-Hui Lv *MD*^2^, Li-Ming Huang *MD*^2^, Wu-Yi You *MD*^6^, Bin Wang *MD*^7^, Kong-Ying Lin *MD*^2,8*^, Alfred Wei Chieh Kow *MD, FRCS Ed, FACS*^9#^, Yong-Yi Zeng *MD*^1,2,8#^

**Table S1.** Univariable and multivariable analysis of risk factors associated with SAR.

**Figure S1.** Flow chart of the study. HCC, hepatocellular carcinoma; BCLC, Barcelona Clinic Liver Cancer.

**Figure S2.** Comparison of survival after recurrence between patients with time to recurrence (TTR) ≤6 months and TTR >6 months in early-recurrent hepatocellular carcinoma, stratified by Barcelona Clinic Liver Cancer (BCLC) stage at recurrence and treatment modality. (A) Patients with BCLC 0/A stage at recurrence; (B) Patients with BCLC B stage at recurrence; (C) Patients with BCLC C stage at recurrence; (D) Patients receiving curative treatments; (E) Patients receiving non-curative treatments.

# **Table S1.** Univariable and multivariable analysis of risk factors associated with SAR.

| **Characteristics** | **HR Comparison** | **UV HR (95% CI)** | **UV *P*** | **MV HR (95% CI)** | **MV *P*** |
| --- | --- | --- | --- | --- | --- |
| **Age** | > 55 *vs*. ≤ 55 years | 0.897 (0.691-1.164) | 0.413 |  |  |
| **Gender** | Male *vs.* female | 1.489 (0.950-2.333) | 0.082 |  |  |
| **HBV infection** | Yes *vs.* no | 1.165 (0.635-2.136) | 0.622 |  |  |
| **Cirrhosis** | Yes *vs*. no | 0.985 (0.692-1.401) | 0.931 |  |  |
| **Child-Pugh grade** | B *vs*. A | 1.605 (1.104-2.334) | 0.013 | *NS* | 0.611 |
| **Portal hypertension** | Yes *vs.* no | 0.795 (0.554-1.140) | 0.212 |  |  |
| **Albumin** | < 35 *vs.* ≥ 35 g/L | 1.342 (1.031-1.747) | 0.029 | 1.465 (1.093-1.964) | 0.011 |
| **Total bilirubin** | > 17.1 *vs.* ≤ 17.1 umol/L | 1.187 (0.913-1.543) | 0.201 |  |  |
| **AFP** | > 400 *vs.* ≤ 400 ng/ml | 2.333 (1.762-3.088) | < 0.001 | 1.453 (1.065-1.981) | 0.018 |
| **Time to recurrence** | ≤ 6 vs. > 6 months | 2.163 (1.665-2.812) | < 0.001 | 1.490 (1.125-1.972) | 0.005 |
| **BCLC** | B *vs*. A | 2.303 (1.637-3.240) | < 0.001 | 1.699 (1.174-2.458) | 0.005 |
| **BCLC** | C *vs*. A | 3.358 (2.490-4.529) | < 0.001 | 2.068 (1.452-2.945) | < 0.001 |
| **Treatment for recurrence** | Non-curative *vs*. curative | 2.971 (2.231-3.956) | < 0.001 | 1.981 (1.430-2.742) | < 0.001 |
| **Tumor size (initial tumor)** | > 5 *vs*. ≤ 5 cm | 1.383 (1.064-1.797) | 0.015 | *NS* | 0.330 |
| **Tumor number (initial tumor)** | Multiple *vs*. single | 1.491 (0.932-2.386) | 0.096 |  |  |
| **Tumor encapsulation (initial tumor)** | Incomplete *vs*. complete | 1.353 (0.981-1.866) | 0.065 |  |  |
| **Tumor differentiation (initial tumor)** | Poor *vs*. well | 1.167 (0.885-1.541) | 0.274 |  |  |
| **Satellite nodules (initial tumor)** | Yes *vs*. no | 1.433 (1.084-1.893) | 0.011 | *NS* | 0.183 |
| **Microvascular invasion (initial tumor)** | Yes *vs*. no | 1.578 (1.132-2.200) | 0.007 | 1.432 (1.015-2.020) | 0.041 |

**Abbreviations:** *AFP*, α-fetoprotein; *BCLC,* Barcelona Clinic Liver Cancer; *CI*, confidence interval; *HBV*, hepatitis B virus; *HR*, Hazard ratio; *MV*, multivariable; *NS*, not significant; *SAR*, survival after recurrence; *UV*, univariable.

# **Figure S1.** Decision tree analysis to determine the optimal cutoff value of time to recurrence for survival after recurrence.

**
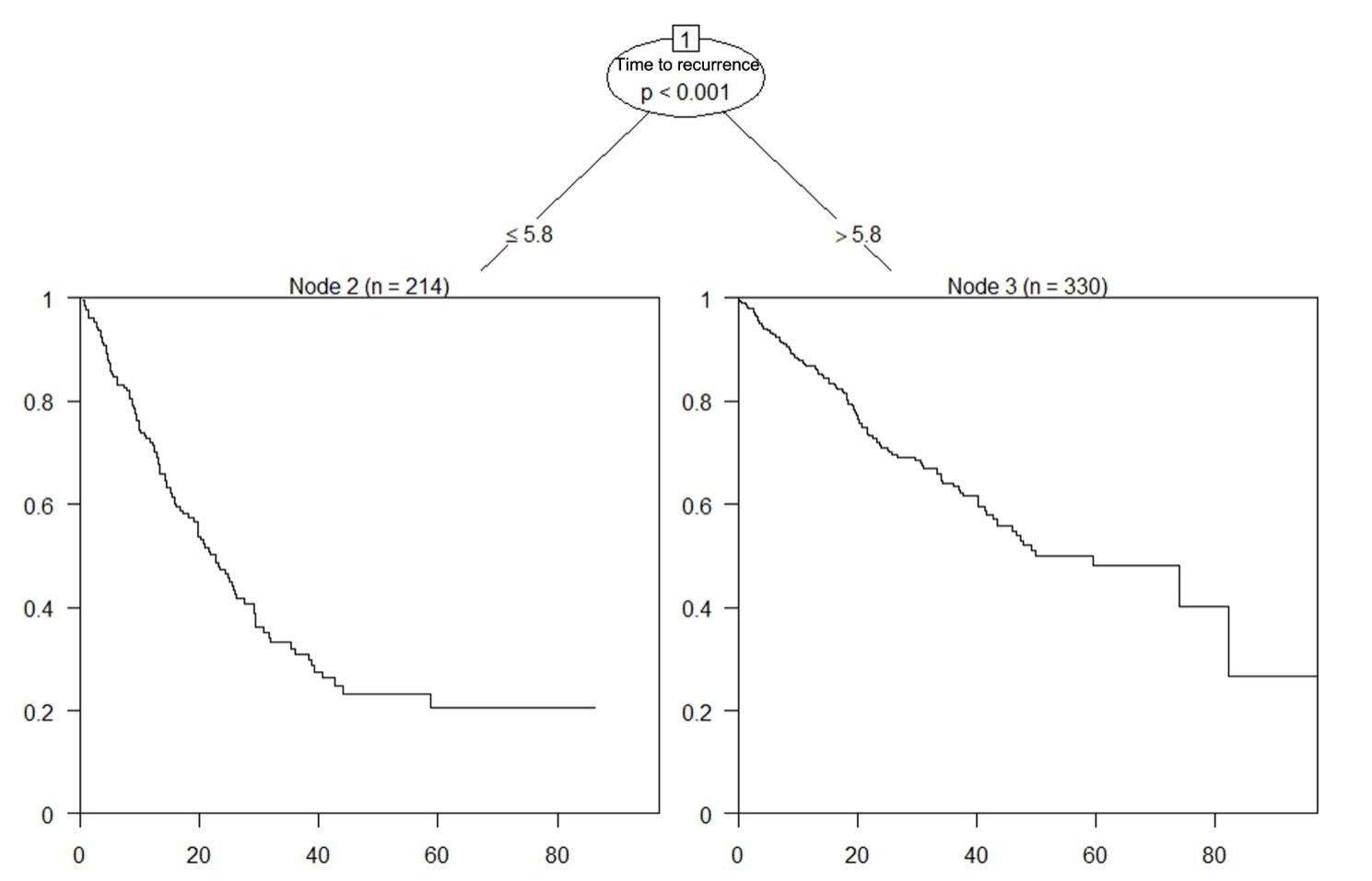
**

# **Figure S2.** Comparison of survival after recurrence between patients with time to recurrence (TTR) ≤6 months and TTR >6 months in early-recurrent hepatocellular carcinoma, stratified by Barcelona Clinic Liver Cancer (BCLC) stage at recurrence and treatment modality. (A) Patients with BCLC 0/A stage at recurrence; (B) Patients with BCLC B stage at recurrence; (C) Patients with BCLC C stage at recurrence; (D) Patients receiving curative treatments; (E) Patients receiving non-curative treatments.


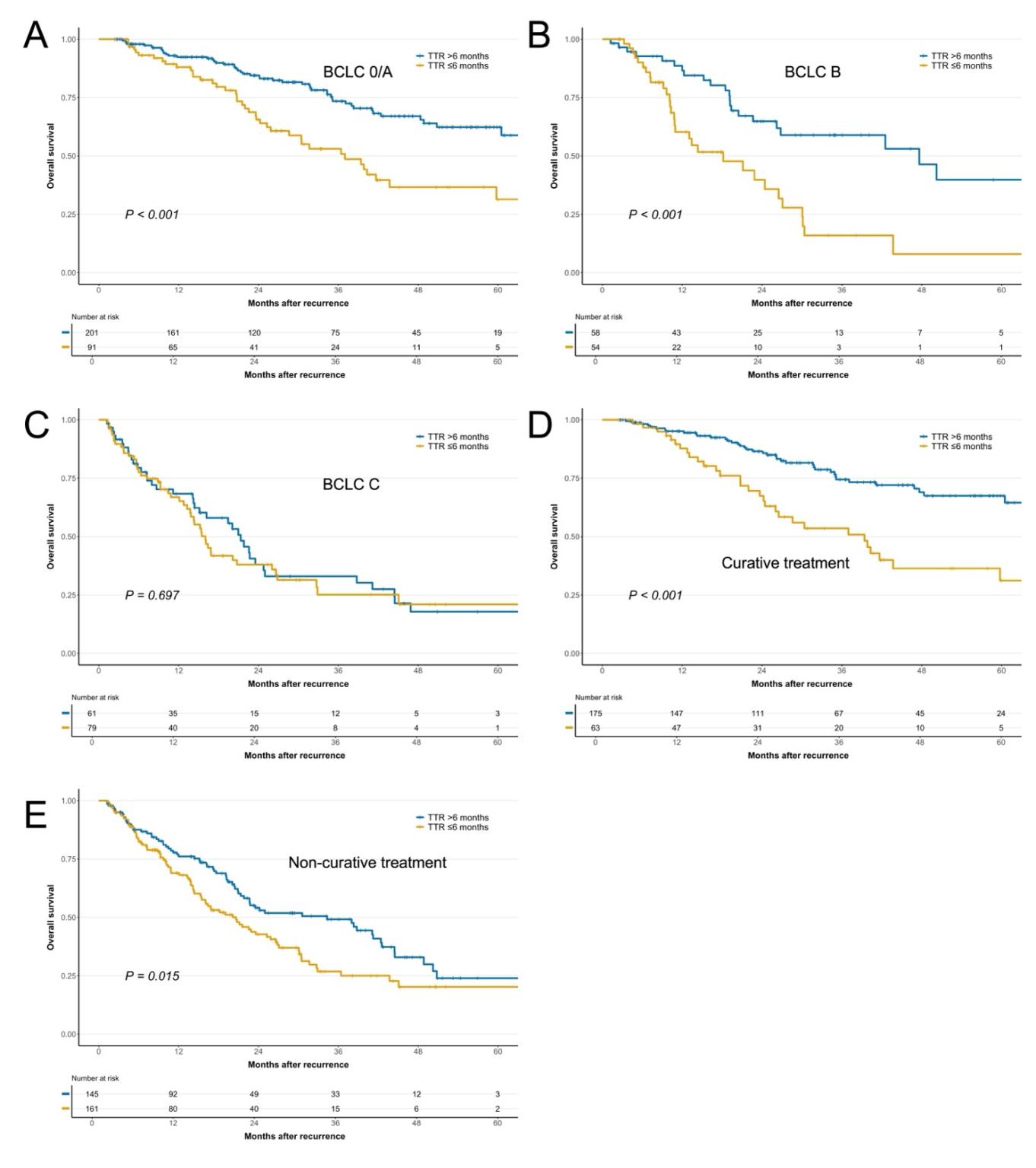

Supplement: Supplementary file 1 [file DataSheet1.docx]
